# Minne Ties Hybrid Arch Bar System vs. Erich Arch Bars: A Cadaveric Comparison Study

**DOI:** 10.3390/cmtr19010007

**Published:** 2026-01-20

**Authors:** Jeffrey Mella, François E. Proulx, Alan W. Johnson

**Affiliations:** 1 Department of Otolaryngology—Head & Neck Surgery, University of Minnesota, Minneapolis, MN 55455, USA; 2 Department of Oral Maxillofacial Surgery, University of Minnesota, Minneapolis, MN 55455, USA; proul076@umn.edu; 3 Hennepin Healthcare, Minneapolis, MN 55415, USA

**Keywords:** maxillomandibular fixation, dental occlusion, mandible fracture, maxilla fracture, hybrid arch bar, surgical efficiency

## Abstract

Jaw fracture management significantly advanced with the introduction of Erich Arch Bars (EABs) during World War II, becoming the gold standard for maxillomandibular fixation (MMF). EABs, however, are time-consuming, pose risks of sharps injuries, and hinder oral hygiene and patient comfort. This study tested the Minne Ties Hybrid Arch Bar System (MTHAB), a novel MMF technology. This cadaveric study used specimens with near-complete dentition to compare MTHAB and EABs. The technologies were applied by trained surgeons to measure occlusal forces, increasing elastic loads, and application and removal times. Surgeons completed structured usability surveys. The results indicated that MTHAB significantly reduced application time (19.8 ± 4.1 min versus 35.2 ± 5.7 min, *p* = 0.0027) and removal time (1.6 ± 0.4 min versus 5.1 ± 2.1 min, *p* = 0.0465) compared to EABs, while also being rated higher for ease of use and safety. Both technologies achieved acceptable occlusion forces, although MTHAB needed more elastics to achieve comparable forces to EABs. While MTHAB appears promising, future clinical trials are needed to evaluate long-term outcomes, fixation stability, and patient selection. MTHAB represents a potential advancement in MMF technology, balancing surgical efficiency, safety, and fixation strength.

## 1. Introduction

Modern management of jaw fractures, including the mandible and maxilla, can be traced back to World War II. “Arch bars”, as described in John B. Erich’s *Traumatic Injuries of Facial Bones: An Atlas of Treatment (1944)* [1], became the standard of treatment and are commonly referred to as Erich Arch Bars (EABs) to this day. Their simplicity of design and versatility in treating the majority of types of mandible fractures have made them indispensable to facial trauma surgeons. They reliably secure the mandibular and maxillary dentition into stable occlusion, which is the definition of maxillomandibular fixation (MMF). MMF can be used as a stand-alone treatment for fractures of the upper or lower jaw (closed reduction) or it can be used in combination with internal fixation techniques, typically involving placing titanium plates and screws directly into the bone for fracture reduction and fixation.

In recent decades, numerous other techniques have emerged to establish MMF. While EABs remain the gold standard, they have multiple limitations including time-consuming application, patient discomfort, limited oral hygiene, and sharps injury risk due to the wires used to apply the bars. These newer techniques, including intermaxillary fixation (IMF) screws [2], embrasure wires [3], hybrid arch bars [4], and dental occlusion ties [5], aim to avoid the limitations of EABs. Numerous recent comparison studies have detailed the key features and limitations of different techniques [6,7,8,9,10,11,12,13]. Kalluri et al. published a systematic review and meta-analysis in 2024, further helping define the differences in technique [14]. Recognizing that the varied techniques all have strengths and weaknesses, other authors have published guides on (1) technique specifics [15], (2) how to select an ideal technique [16], and (3) comparisons of how new technologies compare to older ones [17]. Cornelius et al. have compiled the most comprehensive comparison of hybrid MMF systems to date [18].

While all the newer technologies offer specific advantages, none have fully outperformed EABs. The need remains for a technology with the versatility and strength of EABs and with the speed of application, safety, and patient comfort of newer technologies. Minne Ties Hybrid Arch Bar System^TM^ (MTHAB) was designed with these concepts in mind. This technology utilizes Minne Ties^®^ [5] instead of wires to secure the technology to the dentition. This cadaver study aimed to directly compare the occlusal forces as well as the speed of application and removal of EABs compared to MTHAB.

## 2. Materials and Methods

### 2.1. Study Design

**Figure 1:**
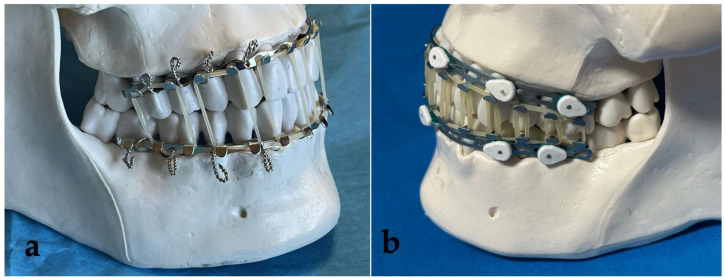
(**a**) Erich Arch Bars applied to a plastic model with 24 g wire with elastic bands to achieve MMF; (**b**) Minne Ties Hybrid Arch Bars applied with circumdental Minne Ties to a plastic model with elastic band MMF.

This cadaver-based experiment was designed to compare and contrast traditional EABs to a novel MMF technology: MTHAB (Invisian Medical, Prairie Village, KS, USA) (Figure 1). To quantify differences in the technologies, the occlusal forces generated by standard elastics bands were assessed. Additionally, the speed of application and removal of the devices were measured. Four fresh cadavers with near complete dentition were supplied by the University of Minnesota’s Anatomy Bequest Program (ABP). A team of 8 surgeons and residents from the University of Minnesota’s Departments of Otolaryngology—Head & Neck Surgery and Oral Maxillofacial Surgery applied the technologies, with 2 participants per cadaver. All surgeons signed agreements to abide by ABP ethical guidelines for cadaver-based studies.

### 2.2. Technology Description

**Figure 2:**
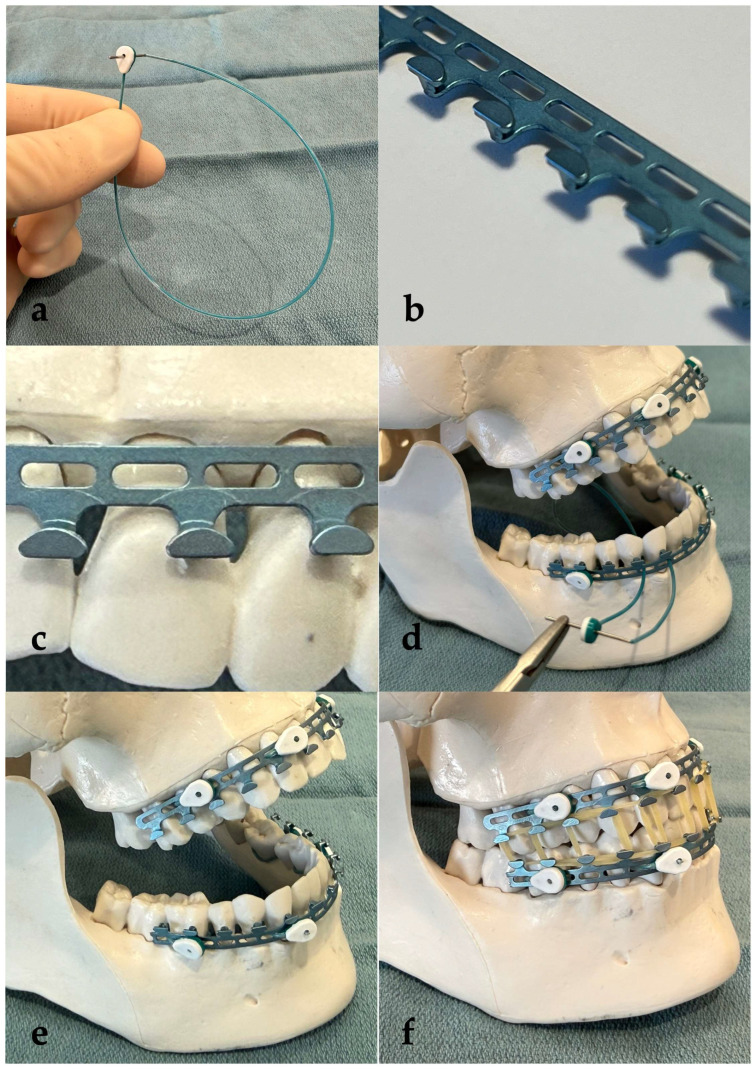
(**a**) Example Minne Tie (dental occlusion tie) which loops upon itself like a “zip tie”; (**b**) MTHAB bar featuring cleat-shaped hooks for elastic band application and ovoid apertures for Minne Ties passage; (**c**) MTHAB positioned along the gingival margin of the teeth; (**d**) a near-complete application of Minne Ties to secure the MTHAB to the model dentition (note the last Minne Tie being applied around the mandibular canine to secure the MTHAB); (**e**) MTHAB fully secured to the dentition (note the Minne Ties have been cut flush with the surface of the Minne Tie clasps to provide a smooth interface with the oral mucosa); (**f**) dentition placed into maxillomandibular fixation with elastic bands applied to the upper and lower MTHAB.

The Minne Ties Hybrid Arch Bar (MTHAB) System (Figure 2) is a novel maxillomandibular fixation (MMF) technology consisting of contoured titanium arch bars secured to the dentition using circumdental Minne Ties (dental occlusion ties) rather than stainless-steel wires. Each bar includes a series of apertures for passage of the Minne Ties and cleat-shaped hooks oriented toward the occlusal surfaces to permit attachment of elastics (or wire loops, if preferred) for MMF. The system is designed to provide versatile MMF optionality including short-term immobilization (up to 4 weeks), or early dynamic functional therapy with guiding elastics. The system streamlines intraoral application by eliminating wire twisting, reducing sharps exposure, and improving access in the mouth during fixation. To apply the technology, the MTHAB bars are trimmed to length, adapted closely to the gingival margins, and secured with Minne Ties placed through aligned interdental embrasures (see Figure 2). Once affixed, elastics can be applied between the superior and inferior bars to achieve maxillomandibular fixation.

### 2.3. Interventions

**Figure 3:**
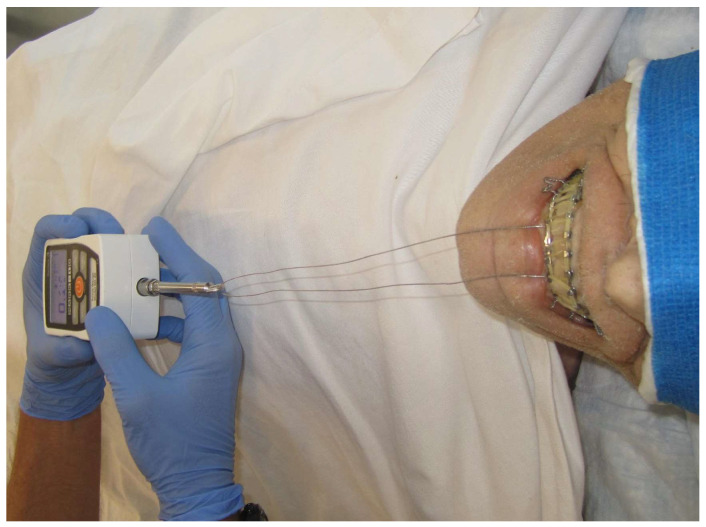
Mark-10 (model M3-50) force gauge applied to mandibular dentition to measure occlusal force achieved by elastic bands.

Each team of 2 surgeons applied EABs and MTHAB to a single cadaver. EABs were applied with 24 g stainless steel wire. A total of 16 circumdental wires were applied. In contrast, the MTHAB was secured with a total of 8 Minne Ties according to the technology’s Instructions for Use. Each application timing was recorded with a stopwatch. Application time was stopped once each technology was fully applied, but before applying any elastic bands. Pictures were obtained with digital cameras before, during, and after application. A complement of elastic bands were applied to each technology, starting with 8 elastics evenly applied to the dentition. The occlusion force was then measured by a force gauge (Mark-10 model M3-50), applied to the mandibular dentition with a single looped 24-gauge wire (Figure 3). The gauge was applied with increasing force until the force overcame the closure force of the elastics, marked by the separation of the maxillary and mandibular dentition. This measurement was then repeated with 10, 12, and finally 14 elastics. Readings were recorded in pounds of force (lb-F).

### 2.4. Endpoints

The primary endpoint was tensile force (lb-F) required to overcome the occlusion achieved by each MMF system. The secondary endpoints included application and removal times, recorded with stopwatches as described above. Additionally, surgeon-reported ease of use, stability, and soft tissue effects were obtained via structured questionnaires using Likert scales.

### 2.5. Equipment and Technology

Standard surgical instruments (retractors, needle drivers, wire cutters, headlamps) were supplied by investigators and the ABP. Erich Arch Bars, Minne Ties Hybrid Arch Bars, Minne Ties, elastics, and the tensile force gauge were provided by Invisian Medical. The experiment was a joint venture between the University of Minnesota and Invisian Medical. Minne Ties were invented at the University of Minnesota [19] and Invisian Medical licenses the intellectual property for commercialization. The Minne Ties Hybrid Arch Bar System (patent pending), is currently not commercially available, with anticipated FDA clearance in 2026.

### 2.6. Statistical Analysis

A linear mixed-effect model was used to compare the tensile forces achieved across the number of elastics. The team was included as a random effect for potential correlation for force measures within a team. Application and removal times were compared between systems using paired *t*-tests. *p*-values less than 0.05 were considered statistically significant. SAS V9.4 (SAS Institute Inc., Cary, NC, USA) was used for the analysis.

## 3. Results

### 3.1. Application and Removal Times

**Figure 4:**
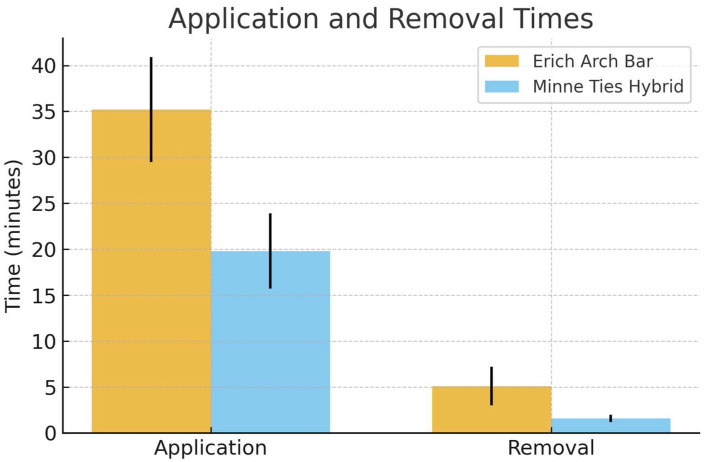
Application and removal times.

Application of the Minne Ties Hybrid Arch Bar was significantly faster than the Erich Arch Bar. The mean application time for the Minne Ties was 19.8 ± 4.1 min compared with 35.2 ± 5.7 min for the Erich Arch Bars (*p* = 0.0027). Similarly, removal was faster with the Minne Ties (1.6 ± 0.4 min) compared with the Erich Arch Bars (5.1 ± 2.1 min, *p* = 0.0465) (Table 1, Figure 4).

### 3.2. Occlusal Force Measurements

**Figure 5:**
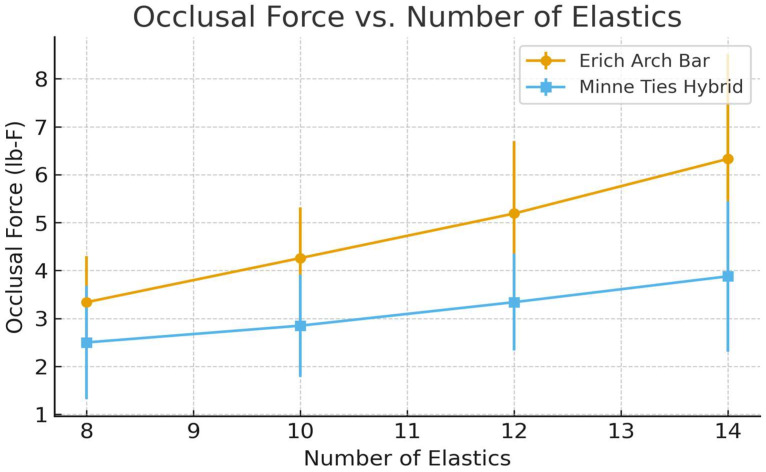
Mean occlusal force (lb-F) by number of elastics for Erich Arch Bar and Minne Ties Hybrid Arch Bar.

Across all elastic conditions, the Erich Arch Bars generated higher tensile occlusal forces compared with the Minne Ties Hybrid Arch Bars. With eight elastics, the mean force for the Erich Arch Bars was 3.34 ± 0.96 lb-F versus 2.50 ± 1.18 lb-F for the Minne Ties. At the maximum of 14 elastics, forces increased to 6.33 ± 2.18 lb-F and 3.88 ± 1.57 lb-F, respectively. A mixed-effect model demonstrated significant main effects for the method and number of elastics (*p* < 0.0001 for both), though no interaction effect was observed (*p* = 0.176) (Figure 5).

### 3.3. Surgeon-Reported Feedback

**Figure 6:**
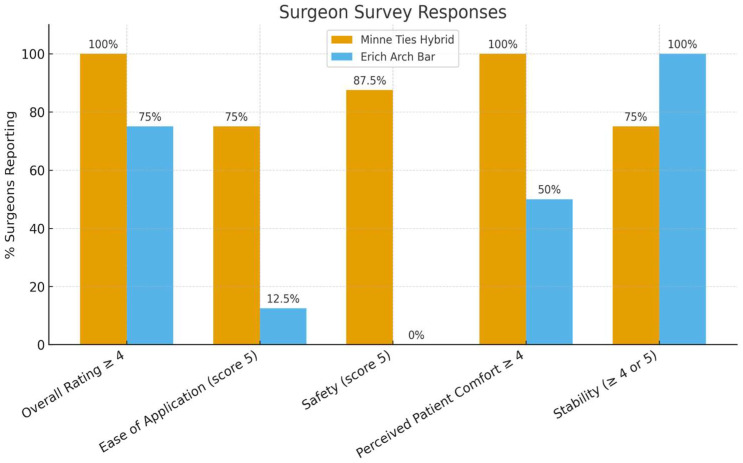
Surgeon survey responses (selected responses).

The results of the Surgeon-Reported Feedback survey can be found in Supplementary Table S1. The survey responses highlighted greater ease of use, improved safety, and equal to improved stability of MMF for the MTHAB system in comparison to EAR. For brevity, the most clinically meaningful survey responses were selected to be highlighted in Table 2 and reflected in Figure 6.

- Overall ratings: 100% of surgeons rated Minne Ties ≥ 4 on a 5-point Likert scale, compared with only 75% for Erich Arch Bars.
- Application ease: 75% of participants rated Minne Ties as “very easy” (score of 5) for applying to dentition, compared with only 12.5% giving Erich Arch Bars the same rating.
- Safety: 87.5% of surgeons rated Minne Ties at the highest safety score (5/5), while Erich Arch Bars scored lower, with 50% rating only 2/5 on safety.
- Stability.

## 4. Discussion

This cadaveric comparison study demonstrates that the Minne Ties Hybrid Arch Bar provides significantly reduced application and removal times compared with traditional Erich Arch Bars, while also offering favorable surgeon-reported perceptions of ease of use and safety.

The findings align with the evolving literature on MMF techniques. EABs, first described in the mid-20th century, remain the benchmark for stability and versatility in mandibular fracture management [1,6,7]. Their primary limitations—time-consuming application, risk of sharps injury, and poor oral hygiene—have motivated the development of alternatives such as IMF screws, embrasure wires, and hybrid systems [6,7,8,10,11]. Studies comparing IMF screws and embrasure wires to EABs have shown improvements in application speed and surgeon ergonomics, though often at the cost of reduced stability or increased risk of complications [7,8,9]. Hybrid systems such as SMARTLock and MatrixWave devices have further narrowed the gap, offering reduced operative time with adequate fixation strength, yet they risk tooth root and nerve injury [9,10,16].

Our results demonstrate that the MTHAB fits into this evolving landscape, providing a pronounced improvement in application efficiency. Application times were nearly halved compared to EABs, a clinically meaningful difference in trauma settings where operative time directly impacts anesthesia exposure and patient outcomes [14,16,17]. Removal was similarly expedited, reducing intraoperative handling and potential for sharps injury. These findings are reinforced by surgeon survey data, which strongly favored MTHAB in terms of ease of application, safety, and perceived patient comfort.

We measured a notable difference between systems in the number of elastics required to generate a given occlusal force: for the same target force, the MTHAB required more elastics than EABs—roughly three elastics for MTHAB for every two with EABs. This pattern is explained by Hooke’s law: the force produced by an elastic increases in proportion to how much it is stretched, and groups of elastics placed in parallel behave like a stiffer spring. Because the distance between the cleats of the superior and inferior bar is smaller for MTHAB than for EAB, each elastic is less elongated at intercuspation and therefore contributes less force per band. Adding more elastics—effectively increasing the overall stiffness—compensates for this geometric difference. Clinically, the key point is that both systems achieved occlusal forces within ranges considered sufficient for maxillomandibular fixation in this model; the MTHAB simply reaches those targets with a higher elastic count.

We also observed meaningful variability in occlusal forces between cadavers at the same nominal elastic count. The vertical height of the dentition varied with each cadaver and influenced the same geometric factor—the distance between the cleats of the superior and inferior bar—which appeared to be the determining variable: greater inter-cleat spacing produced more elastic stretch and therefore higher force per band, regardless of the system. This effect is rarely discussed in the MMF literature but is potentially important for both study design and clinical practice when selecting the appropriate amount of elastics [18].

Several limitations warrant consideration. The study was conducted in a cadaveric model, which while anatomically accurate, cannot replicate intraoral bleeding, tissue compliance, or patient discomfort. The sample size was necessarily limited, and although the use of multiple teams improved generalizability, variability in surgical technique may have influenced the results. Furthermore, MTHAB is not yet commercially available, with anticipated FDA clearance projected for 2026, limiting immediate clinical application.

Designed as a pilot, this study provides a compelling foundation for a planned prospective human-subject randomized clinical trial comparing MTHAB with EABs in the management of mandibular fractures. The trial will evaluate clinical effectiveness—fixation stability, complication rates, oral hygiene, speed of application/removal, and patient-reported outcome measures.

## 5. Conclusions

In summary, the Minne Ties Hybrid Arch Bar System demonstrates substantial advantages in efficiency and surgeon safety compared to Erich Arch Bars, while generating clinically acceptable occlusal forces. The Minne Ties system offers a compelling alternative to traditional methods that may redefine the balance between surgical efficiency, safety, and guiding elastics use in maxillomandibular fixation.

## Figures and Tables

**Table 1:** Application and removal times (minutes, mean ± SD).

**Erich Arch Bars**	**Application Time (min)**	**Removal Time (min)**
Cadaver 1	32.53	5.18
Cadaver 2	35.92	6.03
Cadaver 3	42.85	7.15
Cadaver 4	29.63	2.20
Mean ± SD	35.23 ± 5.7	5.14 ± 2.1
**Minne Ties Hybrid Arch Bars**	**Application Time (min)**	**Removal Time (min)**
Cadaver 1	20.32	1.17
Cadaver 2	22.00	1.33
Cadaver 3	22.83	2.00
Cadaver 4	13.83	1.82
Mean ± SD	19.75 ± 4.1	1.58 ± 0.4

**Table 2:** Surgeon survey responses (% reporting top ratings). Responses are percentages of surgeons rating ≥ 4 or 5 on a 5-point Likert scale (Figure 6).

Category	Minne Ties Hybrid Arch Bar	Erich Arch Bar
Overall Rating ≥ 4	100%	75%
Ease of Application (score 5)	75%	12.5%
Safety (score 5)	87.5%	0%
Perceived Patient Comfort ≥ 4	100%	50%
Strength/Stability of MMF	75%	100%

Responses are percentages of surgeons rating ≥4 or 5 on a 5-point Likert scale.

## Data Availability

The raw data supporting the conclusions of this article are recorded in hard copy in laboratory notebooks and will be made available by the authors on request.

## Supplementary Materials

The following supporting information can be downloaded at https://www.mdpi.com/article/10.3390/cmtr19010007/s1: Table S1: Surgeon-Reported Feedback survey results.

## Abbreviations

The following abbreviations are used in this manuscript:

MMF	Maxillomandibular fixation
ABP	Anatomy Bequest Program
EABs	Erich Arch Bars
MTHAB	Minne Ties Hybrid Arch Bar System
