# Supplementary material for: Minne Ties Hybrid Arch Bar System vs. Erich Arch Bars: A Cadaveric Comparison Study
Source: Craniomaxillofac Trauma Reconstr. 2026 Jan 20;19(1):7. doi: 10.3390/cmtr19010007 (PMC12922075; doi:10.3390/cmtr19010007)
Supplement: Supplementary file 1 [file cmtr-19-00007-s001.zip › cmtr-3989066-supplementary.pdf]

Supplementary Table S1. Complete Surgeon Survey Responses (Values are percentages by Likert category).

Question	System	1 (%)	2 (%)	3 (%)	4 (%)	5 (%)
Ease of Forming to Arch	Minne Ties Hybrid	0	0	25	25	50
Ease of Forming to Arch	Erich Arch Bar	0	0	0	75	25
Ease of Cutting to Length	Minne Ties Hybrid	0	0	12.5	12.5	75
Ease of Cutting to Length	Erich Arch Bar	0	0	12.5	12.5	75
Ease of Applying to Dentition	Minne Ties Hybrid	0	0	0	25	75
Ease of Applying to Dentition	Erich Arch Bar	0	25	62.5	12.5	0
Total Application Time	Minne Ties Hybrid	0	0	0	25	62.5
Total Application Time	Erich Arch Bar	12.5	50	25	0	0
Your Safety	Minne Ties Hybrid	0	0	0	12.5	87.5
Your Safety	Erich Arch Bar	0	50	50	0	0
Access to Hooks - Wire	Minne Ties Hybrid	0	0	25	37.5	37.5
Access to Hooks - Wire	Erich Arch Bar	0	25	12.5	50	12.5
Access to Hooks - Elastics	Minne Ties Hybrid	0	0	37.5	50	12.5
Access to Hooks - Elastics	Erich Arch Bar	0	25	37.5	25	12.5
Strength/Stability of MMF	Minne Ties Hybrid	0	0	25	75	0
Strength/Stability of MMF	Erich Arch Bar	0	0	0	37.5	62.5
Ease of Removal Wire Loops	Minne Ties Hybrid	0	0	0	25	75
Ease of Removal Wire Loops	Erich Arch Bar	0	25	37.5	37.5	0
Ease of Removal Elastics	Minne Ties Hybrid	0	0	25	37.5	37.5
Ease of Removal Elastics	Erich Arch Bar	0	0	25	62.5	12.5
Ease of Removal Bars	Minne Ties Hybrid	0	0	0	12.5	87.5
Ease of Removal Bars	Erich Arch Bar	0	25	62.5	12.5	0
Perception of How Invasive	Minne Ties Hybrid	0	0	0	25	75
Perception of How Invasive	Erich Arch Bar	12.5	12.5	75	0	0
Perception of Patient Comfort	Minne Ties Hybrid	0	0	0	50	50
Perception of Patient Comfort	Erich Arch Bar	25	62.5	12.5	0	0
